# Does brain size affect mate choice? An experimental examination in pygmy halfbeaks

**DOI:** 10.1093/beheco/arab046

**Published:** 2021-08-23

**Authors:** Rebecca M McNeil, Alessandro Devigili, Niclas Kolm, John L Fitzpatrick

**Affiliations:** Department of Zoology: Ethology, Stockholm University, Svante Arrhenius väg 18b, 106 91 Stockholm, Sweden

**Keywords:** brain morphology, decision-making, good genes, male quality, sexual selection

## Abstract

Choosing a mate is one of the most important decisions in an animal’s lifetime. Female mate choice is often guided by the presence or intensity of male sexual ornaments, which must be integrated and compared among potential mates. Individuals with greater cognitive abilities may be better at evaluating and comparing sexual ornaments, even when the difference in ornaments is small. While brain size is often used as a proxy for cognitive ability, its effect on mate choice has rarely been investigated. Here, we investigate the effect of brain size on mate preferences in the pygmy halfbeak *Dermogenys collettei*, a small freshwater fish that forms mixed-sex shoals where mating takes place. Pygmy halfbeaks are ideal models as their semi-transparent heads allow for external brain measurements. After validating the use of external measurements as a proxy for internal brain size, we presented females with large or small brains (relative to body length) with two males that had either a large or small difference in sexual ornamentation (measured by the total area of red coloration). Unexpectedly, neither total relative brain size nor relative telencephalon size affected any measured aspect of mate preference. However, the difference in male sexual ornamentation did affect preference, with females preferring males with a smaller area of red coloration when the difference in ornaments was large. This study highlights the complexities of mate choice and the importance of considering a range of stimuli when examining mate preferences.

## Teaser

Does a bigger brain make you better at choosing a good mate? Using a small fish called a pygmy halfbeak, we show that females with bigger brains are not better at choosing mates compared to females with smaller brains. However, we discovered that female preference depends on how different males are—females prefer males with less red coloration on their bodies when there is a big difference between the two males.

## Introduction

Choosing a mate is one of the most crucial decisions individuals have to make, as it directly impacts their fitness (Ryan et al. 2009). Females typically invest more into each reproductive event and have a lower reproductive rate compared to males, and consequently female mate choice typically defines mating occurrences (Bateman 1948; Trivers 1972). Females commonly choose mates based on the presence or intensity of sexual ornaments. Ornaments can exploit sensory biases or result from runaway selection on arbitrary traits (Andersson 1994; Kokko et al. 2003; Cotton et al. 2004). One of the most common sexual ornaments involves coloration, with preferences commonly shown for conspicuous males (Ryan and Keddy-Hector 1992). However, females exhibit plasticity in their mate choice decisions (Jennions and Petrie 1997), which is hypothesized to maintain within-population variation in sexual ornamentation, as it affects both the strength and direction of selection (Brooks and Endler 2001).

To select a mate, the choosing individual must identify, integrate and compare several cues from multiple individuals (Ryan et al. 2007). Individuals with greater cognitive abilities may be at a selective advantage, provided they can better process these cues, making superior or faster choices. Yet, few studies have examined how cognitive ability and brain morphology influences female mate choice. Cognitive ability is often linked to brain size (Striedter 2005; Kotrschal et al. 2013; Buechel et al. 2018; Striedter and Northcutt 2020), allowing for a simple method to compare cognitive ability both between and within species (Sol et al. 2005; Kotrschal et al. 2013; van der Bijl et al. 2015). Brain size can vary widely within populations (Kotrschal et al. 1998; Striedter 2005; Gonda et al. 2013). As brain tissue is energetically expensive to maintain (Mink et al. 1981; Laughlin et al. 1998), larger brains are commonly assumed to confer fitness advantages. Extensive research on selection lines of large- and small-brained guppies (*Poecilia reticulata*) with associated differences in neuron number (Marhounová et al. 2019) has found that large-brained individuals have multiple advantages: they outperform smaller brained individuals in numerical learning assays (Kotrschal et al. 2013); have increased survival (Kotrschal, Buechel, et al. 2015); improved associative learning (Kotrschal, Corral-Lopez et al. 2015; Buechel et al. 2018); and have a more proactive personality-type (Kotrschal et al. 2014). Additionally, large-brained females are better at distinguishing attractive males in comparison to small-brained females (Corral López et al. 2017). Together, these studies confirm the suggested link between brain size, cognitive ability, and mate choice in one species. Further evidence for the link between cognitive ability and mate choice—but not brain size—has been presented in three-spined sticklebacks (*Gasterosteus aculeatus*): females that spend more time assessing mates make fewer errors in a cognitive learning task (Rystrom et al. 2019). Conversely, Culumber et al. (2020) found that, in the coercively mating eastern mosquitofish (*Gambusia holbrooki*), large-brained females did not successfully reduce male copulation attempts more than small-brained females—in fact, the opposite was the case. Thus, as the only study to find a link between brain size and female mate choice was carried out on artificial selection lines (Corral-López et al. 2017), it remains unclear if standing intraspecific variation in brain size shapes mate choice.

This study investigates whether variation in brain size affects comparative mate preference in pygmy halfbeaks, *Dermogenys collettei*, using a laboratory population of fish descendants from a wild-caught Malaysian population. Pygmy halfbeaks are small, live-bearing, internally fertilizing fish that are native to freshwater areas in south-east Asia (Meisner 2001; Greven 2010). Halfbeaks live in mixed-sex shoals ranging from 6 to 128 individuals, and males in shoals spend nearly one third of their time directing courtship behaviors toward females (Devigili et al. 2021). Halfbeaks are sexually dichromatic, with males displaying strong red coloration that females use to exert mating preferences (Reuland et al. 2019). However, female preference is influenced by mating status in a counter-intuitive fashion: previously mated females prefer males with a large amount of red, whereas virgin females prefer males with a small amount of red (Reuland et al. 2019). Halfbeaks also have semi-transparent heads, with a clear dorsal outline of the brain visible through the skull (including the dorsal outline of the optic tectum and telencephalon), allowing for noninvasive external estimates of both total brain size and the size of individual brain areas (sensu Näslund 2014). Combined, these factors make the pygmy halfbeak an ideal candidate for assessing the outcome of brain size variation on female mate preference for sexual ornaments using a noninvasive measurement technique. To this end, we first validate that external measures of brain size predict internal brain size measurements obtained following dissection. We then use a dichotomous choice test to experimentally assess whether brain size affects comparative female mate choice behavior when presented with two males that exhibited either a large or small difference in their area of red coloration. Previous studies have found that mate preference can often vary dependent on the set of options presented (MacLaren and Rowland 2006; Sato et al. 2014; Wacker et al. 2016). We hypothesized that female preference will be stronger in large-brained females when the difference in red coloration between the stimuli males is small, as this should present a greater cognitive challenge. Both female responsiveness (the time a female spends assessing males) and the number of times a female switches between males are hypothesized to be lower in large-brained females as they should be able to process information and make choices faster.

## MATERIALS AND METHODS

### Study animals and rearing conditions

Fish were obtained from two sources, which vary depending on the question being addressed (designated as Part 1 and 2 below). To investigate the use of external brain measurements as a proxy for internal brain size (Part 1), descendants of commercially sourced individuals were sampled (Ruinemans Aquarium B.V., Montfoort, The Netherlands). To investigate female mate preference (Part 2), a mix of F2 and F3 descendants of wild-caught halfbeaks sourced from the Tebrau river, Malaysia were used. The aim of Part 1 of the study (i.e., linking external and internal brain measurements) required fish be euthanized and dissected. Therefore, we used commercially sourced fish rather than wild-caught fish as the former can be obtained with comparative ease. Importantly, barcoding using cytochrome oxidase submit I (COI) demonstrates that the commercially sourced fish used in Part 1 of this study are distributed throughout the cluster of wild-caught fish used in Part 2 of this study (N. Puniamoorthy, A. Devigili, E. Fernlund Isaksson and J.L. Fitzpatrick, unpublished data).

All fish were reared in the laboratory using a standard set of husbandry practices. All aquaria were oxygenated and contained ~2 cm of gravel and a mixture of both live and artificial plants. Gravid females were kept in 7.5 L tanks and monitored daily. Upon giving birth, offspring (i.e., fry) were separated from mothers to prevent maternal cannibalism and kept in 5 L tanks with up to five other fry. At the onset of sexual maturity, males and females were placed either into mixed sex (Part 1) or sex-specific (Part 1 and 2) 50 L or 160 L tanks. The onset of sexual maturity was determined by identifying maturing males by their thickened andropodium, a fused set of five fin rays that are used to transfer sperm to the female. Fish age varied between 4 and 24 months. A 12:12 dark/light cycle was maintained within the laboratory and the temperature was kept at 26 ± 2 °C. Fish were fed a mix of flake food and recently hatched *Artemia*. All experiments were conducted in accordance with the Stockholm Animal Research Ethical Board (permit number 2393-2018 and 3867-2020).

### Part 1: External brain measurements as a proxy for internal brain size

#### External measurements of brain morphology

**Figure 1:**
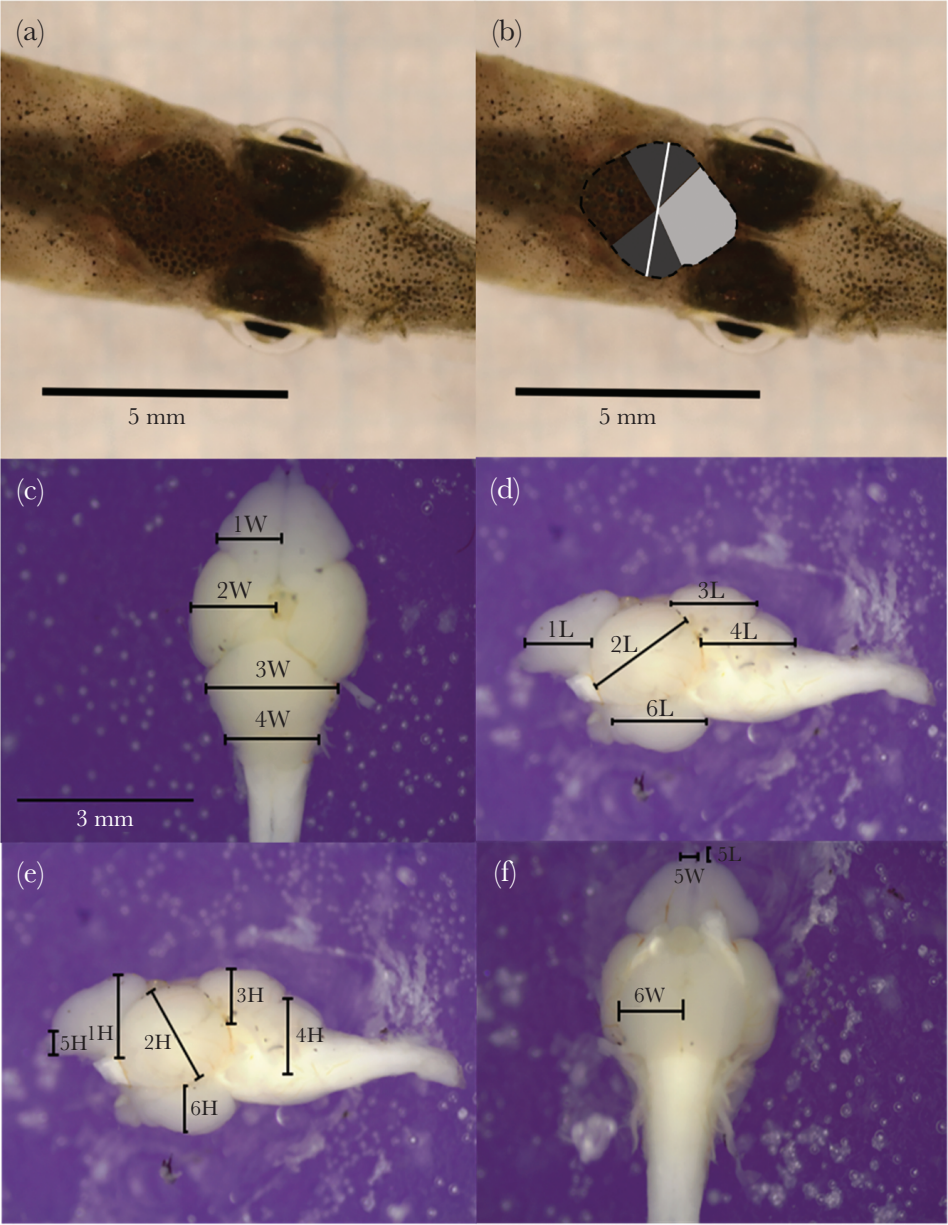
Brain measurements. External (a, b) and internal (c, d, e, f) perspectives of the brain. In (a), an external view of an unmeasured brain is shown. In (b) all measurements taken of the external brain are presented, including: total brain area (dotted black line); brain width (solid white line); telencephalon area (light gray area); optic tectum area (dark gray area). In c–f measurements of the width (W), height (H), and length (L) for brain structures measured using dorsal (c), lateral (d+e), and ventral (f) photographs are shown. The 6 measured structures were as follows: (1) telencephalon; (2) optic tectum; (3) cerebellum; (4) dorsal medulla; (5) olfactory bulbs; (6) hypothalamus. Photo credit: R.M. McNeil and A. Devigili.

Pygmy halfbeaks have semi-transparent heads that allow internal structures to be visualized on live fish. Taking advantage of their semi-transparent heads, a simple, noninvasive technique to externally measure brain morphology in adult female pygmy halfbeaks was developed by modifying the methods described in Näslund (2014). External measurements were taken of the dorsally visible brain and individual brain areas, including total dorsal brain area and width, and dorsal telencephalon and optic tectum area for *n* = 32 females (Figure 1a,b). Dorsal photographs were also used to measure the standard length of the fish (from the tip of the snout to the caudal peduncle). Total brain measurements and, where possible, individual brain region areas were measured. Brain regions were traced using ImageJ v1.52q (Schneider et al. 2012). The skull represents a dark, shaded area in the centre of the head (Figure 1a). The total dorsal brain area was defined as the entire shaded area of the skull, which encompassed the olfactory bulbs, telencephalon, optic tectum, cerebellum, and dorsal medulla (Figure 1b). The dorsal width of the brain was defined as the largest distance enclosed by the skull in the left-right axis (Figure 1b). Internally, this width corresponds to the width of the optic tectum. Of the individual brain regions, only the dorsal area of the telencephalon and optic tectum could be measured externally, as their boundaries were clearly defined through the skull (Figure 1b). The area of the telencephalon encompassed the entire front section of the brain, anterior to the optic tectum. The transition from the optic tectum to the telencephalon had a distinct “V” shape (Figure 1a–c). The outer edge of the optic tectum was evident from a circular bulge in the skull. At the posterior end of the optic tectum, the transition to the cerebellum had a distinct inverted “V” shape (Figure 1a–c).

All external brain values were measured by a single researcher (R.M.M.). To assess whether these external measurements were repeatable between observers, a newly trained observer re-measured total external dorsal brain area and width, and these values were statistically compared to the values measured by R.M.M. The repeatability of the external measurements between observers was significant and high for measurements of dorsal brain area (R = 0.77 [0.50–0.93] confidence interval [CI], likelihood ratio test [LRT] = 0.002) and dorsal brain width (R = 0.77 [0.25–0.91], LRT = 0.002).

#### Internal measurements of brain morphology

After fish were photographed, they were euthanized in an overdose of benzocaine and fixed in 4% phosphate-buffered formalin for 3–5 days. Fish were washed twice in phosphate-buffered saline (PBS): the duration of the first wash was 1 day and the duration of the second wash ranged from 1 to 5 days.

Brains were dissected out under a Leica S9i stereo microscope (Leica Microsystems Ltd., Heerbrugg, Switzerland), with fine scale dissection equipment. The brain was cut where the spinal cord enters the brain stem. Once removed, brains were placed in a small petri dish containing solid dark candle wax and PBS solution. Dorsal, right lateral, left lateral, and ventral photographs were taken using the dissection microscope’s inbuilt 10-megapixel CMOS camera with associated Las X software (Leica Microsystems Ltd.). A scale was photographed following each brain in order to later calibrate the pictures. Brains were then blotted dry and weighed on a scale to the nearest 0.01 mg (Balance XS105, Mettler Toledo, Columbus, OH, USA). ImageJ was used to calculate precise measurements of the height, width and length of each individual brain area (Figure 1c–f). Following the method of Pollen et al. (2007), the volume of each brain area was calculated according to an ellipsoid model using V=(LxDxH) x π/6. All structures were measured from both the right and left side, in addition to dorsal and ventral measurements. To calculate the total volume of paired brain areas, such as the optic tectum, both sides were summed. In the rare case that a value was missing for one side (*n* = 2), the measured value was doubled as structures are generally symmetrical. To calculate the volume of unpaired structures, the final value of each measurement was taken as the average of both sides. When a value was missing from one side (*n* = 1), the value of the measured side was taken as the final value. For each brain, the volume of each structure was summed to give the total internal brain volume.

Although 32 fish were measured, in one case a brain region was damaged during dissection and this fish was removed from the analysis. Additionally, one fish had a particularly large brain for its body size that fell significantly outside the line of best fit and disproportionally influenced the allometric relationship between brain and body size. Therefore, this fish was also removed from the analysis. This gave a final sample size of 30.

### Part 2: Brain size and female mate preference

Dichotomous choice assays were used to assess whether females with large brains differed in aspects of mate preference when compared to females with small brains. All females were presented with a pair of males in a dichotomous choice chamber; pairs either had a larger difference in red area on their fins and beak or a smaller difference in red area. The amount of red coloration on males is the main (and seemingly only) visual factor influencing female preference in pygmy halfbeaks during dichotomous trials (Reuland et al. 2019). Experiments were carried out in two blocks, and classifications of female brain size (i.e., large vs. small) were determined for each block separately. However, standardized values of brain area were qualitatively similar between each block.

Female pygmy halfbeaks exhibit mating status-dependent preferences, with mated females preferring males with *more* red coloration and virgin females preferring males with *less* red coloration in dichotomous choice assays (Reuland et al. 2019). Reuland et al. (2019) suggested that one possible explanation for these mating status-dependent preferences was that virgin females, who were reared in all-female aquaria, were naive to males and sexually inexperienced, which may have influenced their mating preferences. To address this potential explanation, we allowed virgin females to interact with males prior to the experiment, while maintaining their virgin mating status. Specifically, three virgin females and three virgin males were placed in 55 cm diameter arenas filled with ~6 cm of water and allowed to interact for 6–8 h. During this time, we observed males courting and attempting to mate with females. However, as males must swim under females when performing courtship behaviors and must twist their body rapidly to transfer sperm to females when attempting to mate (Greven 2010), we hypothesized that maintaining fish in shallow (~6 cm) water would prevent males from successfully transferring sperm to females. Indeed, following the dichotomous choice trials described below, none of the females gave birth when maintained in isolated tanks for three months (equaling roughly three brood cycles). Therefore, we consider the females used in this study as virgins, but sexually experienced.

#### Generating “large” and “small” brained female treatment groups

Females used in the dichotomous choice assays were photographed dorsally (as in Part 1) and their external total dorsal brain area and width, dorsal telencephalon and optic tectum area, and standard length were measured as in Part 1. Eighty-one females were measured in each block. Residual values were calculated from a linear regression of external total dorsal brain area fit against standard length within each block and the upper 30% (*n* = 23 in block 1 and *n* = 24 in block 2) and lower 30% (*n* = 23 in block 1 and *n* = 24 in block 2) were categorized as “large” and “small” brained females, respectively. Using this method, all brain sizes were classified relative to body length. Henceforth, this categorical variable of external brain size relative to body length (large vs. small) is referred to as “total relative external brain area.” This gave a total of 94 females (*n* = 46 and *n* = 48 in block 1 and 2, respectively, equally divided between large- and small-brained females). Total relative external brain area differed on average by 11.6% between the large and small female categories when both blocks were combined.

#### Quantifying male sexual ornaments

**Figure 2:**
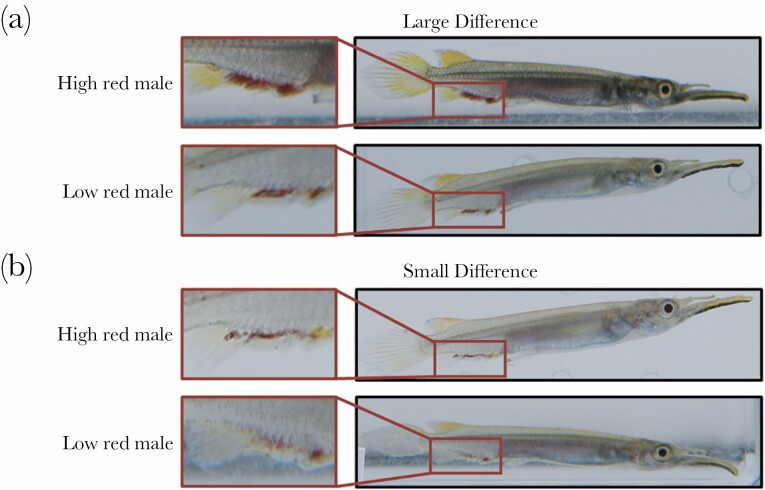
Male dyad treatments. Examples of male dyads (pairs) presented to females in a dichotomous choice chamber. Dyads were created to form two treatments based on the magnitude of difference in male red ornamentation: (a) the large difference treatment, where males differed in red area by 0.4–0.8 mm^2^ (average ± SE: 0.59 ± 0.02 mm^2^); (b) the small difference treatment, where males differed in red area by 0.1–0.3 mm^2^ (average ± SE: 0.20 ± 0.01 mm^2^). Photo credit: R.M. McNeil and A. Devigili.

Sixty-one males were sampled in block 1 and 93 males were sampled in block 2. Males were photographed laterally in order to measure standard length and red area using ImageJ. The total red area was determined by tracing around visible red markings on the beak and fins, resulting in a total red area measurement in mm^2^. Within blocks, males were then paired to form dyads where the difference in total red area between males was classified as either a “large difference” (between 0.4 and 0.8 mm^2^) (Figure 2a) or a “small difference” (between 0.1 and 0.3 mm^2^) (Figure 2b). In total, 45 male dyads were produced, using 90 of the sampled males. The difference in red coloration between the stimuli males was nearly three times greater in the “large difference” dyads (average ± SE: 0.59 ± 0.02 mm^2^) than the “small difference” dyads (0.20 ± 0.01 mm^2^). As expected, the difference in red coloration between stimuli males in the large and small difference groups was statistically different (*t*-test; *t* = 20.98, *P* < 0.01). However, the total amount of red coloration present on males in the large and small difference treatment groups did not differ from one another (*t*-test = 1.13, *P* = 0.26). Within these dyads, the male with a larger area of red was classed as the “high red male” and the male with a smaller area of red was classed as the “low red male.” In the “large difference” treatment, the average ± SE “high red” male had a red area of 0.70 ± 0.03 mm^2^ and the “low red” male had a red area of 0.1 ± 0.04 mm^2^. In the “small difference” treatment, the average “high red” male had a red area of 0.43 ± 0.03 mm^2^ and the average “low red” male had a red area of 0.24 ± 0.03 mm^2^. Males with no red were excluded since our aim was not to investigate the effect of the presence versus absence of sexual coloration on female behavior. Males were size-matched (± 3 mm) within each dyad, although body size was not expected to affect female choice based on previous findings (Reuland et al. 2019). The majority of male dyads were used twice on the same day, and dyads were used in up to six trials, although never on multiple days in a row. Trials including one male dyad (*n* = 2) were excluded, as this dyad had a difference in total red area of 1.25 mm^2^, which was considerably higher than the average difference in red area in the “large difference” dyads.

#### Dichotomous choice experiment

Dichotomous choice chamber tanks (45 cm × 25 cm × 20 cm) were used to assess female preference. Tanks consisted of a main chamber (45 cm × 15 cm × 20 cm) which housed the focal female, and three smaller chambers (each 15 cm × 10 cm × 20 cm). The middle chamber did not contain a male but was used as a neutral zone and spatial separator between the two stimuli males placed in the side chambers. Each chamber was filled with ~5 cm of water and ~1 cm of white gravel. A transparent glass wall separated stimuli males from the focal female to prevent olfactory cues but allow visual cues for assessment. Opaque dividers surrounded the tank to prevent visual contact with the outside environment. Opaque acrylic dividers separated stimuli males to prevent visual contact. The placing of males was randomized in each trial to account for any effects of learning or side-biases. Fish were given a 45-min period of habituation, in which visual and chemical cues between the focal female and stimuli males were prevented by an opaque divider. After this period, the divider was removed via a pulley system to minimize disturbance to the fish, introducing visual (but not chemical) cues to the female. Trials lasted a total of 60 min and were filmed using GoPro Hero 5 Black Digital Cameras (GoPro, Inc., San Mateo, CA, USA) placed 25 cm above the tank. Videos were later analyzed using Behavioural Observation Research Interactive Software (BORIS) (Friard and Gamba 2016), with video analyses being performed by a researcher (R.M.M.) blind to both female category and male dyad treatment. The length of time the focal female spent in each males’ association zone was recorded for each trial. Association time is regularly used as a proxy measure of female mate choice (Walling et al. 2010; Dougherty 2020). Female responsiveness was measured by calculating the total time a female spent in both male association zones combined. The number of switches was calculated by determining how many times a female moved from one association zone to the other. Responsiveness is typically a measure of the energy, time and/or interest the female allocates to mate choice (Jennions and Petrie 1997; Rystrom et al. 2019). In this study, responsiveness represents the amount of time a female takes to decide between the stimuli males. Once this decision is made, no further sampling should be necessary, creating lower values of responsiveness. Females with bigger brains are predicted to make these decisions faster, and hence have lower responsiveness values. Similarly, a lower number of switches was also used as a proxy for sampling time and a measure of indecisiveness, assuming that once a female chooses a stimuli male, she would no longer switch between males. Again, females with larger brains are predicted to decide faster and therefore switch fewer times between stimuli males. The magnitude of difference between male red area is also likely to affect both responsiveness and number of switches—males which have a large difference in red are predicted to be easier to discriminate, thus allowing the female to choose a mate faster, leading to lower responsiveness and lower number of switches. Each focal female was used twice, with trials occurring directly after one another. After each trial, all water was removed to prevent olfactory cues from influencing later fish. The fish were given the same 45-min period of habituation before the second trial. All females were exposed to both large and small male difference treatments, with the order of presentation randomized.

Informed choice could only occur after the female had visited the association zone of both males, and hence association times were only statistically investigated if the female visited the second male’s association zone. Trials were then scored for 30 min. Some females visited the association zone of the second male late into the trial and hence the duration of informed choice was less than 30 min. If the duration of informed choice was less than 10 min, the trial was excluded. A total of 188 trials were filmed. However, 20 trials were excluded for various reasons: the focal female never visited both males (*n* = 11), the focal female never visited either male (*n* = 2), the duration of informed choice was less than 10 min (*n* = 2), or due to experimenter error (*n* = 5). In effect, this meant that 20 females were either removed completely or only considered in one of the experimental treatments during subsequent statistical analyses. Thus, the final sample size used in analyses was a total of 168 trials, with roughly equal numbers of large-brained (*n* = 47) and small-brained females (*n* = 43) tested. As the order of male presentation was randomized, a roughly equal number of large-brained females experienced a large difference dyad followed by a small difference dyad (*n* = 24) compared to a small difference dyad then a large difference dyad (*n* = 23). Similarly, roughly equal numbers of small-brained females experienced a large then small difference dyad (*n* = 22) compared to a small then large difference dyad (*n* = 21).

### Statistical analyses

#### Part 1: External brain measurements as a proxy for internal brain size

All analyses were carried out on RStudio v1.2.5019 (Rstudio Team 2019). To compare external brain measurements with internal brain measurements all values were standardized by subtracting the average value of each variable and dividing by the standard deviation. This was necessary as measurements were taken on different scales (i.e., area, volume and weight). Using these standardized values, the Rstudio package “rptR” (Stoffel et al. 2017) was used to estimate the pairwise repeatability between each external and internal measure. This method calculates a repeatability measure, R, by calculating the variance among group means (group level variance) over the sum of the group-level plus data-level variance (residual variance). Parametric bootstrapping was used to estimate the uncertainty in the estimated R value. The number of parametric bootstraps was set to 100 and this was kept constant for all repeatability tests. Pairwise repeatability tests were also used to test the repeatability between external telencephalon area and internal telencephalon volume and the repeatability between external optic tectum area and internal optic tectum volume. The repeatability of external measures of brain area and width between observers was assessed using the raw data.

#### Part 2: Brain size and female mate preference

To assess female preference while accounting for the bounded nature of the response variable (i.e., values ranged from zero to one), generalized linear mixed effects models (glmers) were used with a binomial error distribution fitted with a logit function using R Studio package “lme4” version 1.1–23 (Bates et al. 2015). For all glmer models, the optimizer was set to *bobqa* and overdispersion was assessed using the *overdisp_fun* function. Initial glmer models investigated main and interactive effects and included female identity (to account for repeated measures of females), male dyad identity (to account for repeated use of male dyads) and observation number (to account for overdispersion) added as random effects. However, these initial models routinely suffered from poor model fits, were characterized by singular fits, and were overdispersed. Model diagnostics revealed that the random effects were responsible for the poor overall fit of these initial models. Approaches for dealing with poorly fit random effects remain controversial. Specifically, the inclusion of poorly fit (i.e., models with singularities) random effects into our modeling approach would acknowledge the repeated use of females and male dyads in the experiment. However, if the inclusion of random effects leads to overdispersed models the results are unlikely to be robust. To improve model fits and minimize the effects of overdispersion, we chose to sequentially remove male dyad identity, followed by female identity as random effects in cases where model fits were poor to allow us to focus on better fitting, albeit simplified, models. We specify which random effects were removed from each model below.

We began by assessing if females demonstrated a preference for one male over the other throughout the observation period. To do this, we used the *cbind* function in R to bind “time with more-preferred male” with “time with less-preferred male,” which was then treated as the dependent variable in a model fitted without the intercept, with observation number added as a random effect (note that female identity and male dyad identity were excluded as their inclusion led to a singularity in the model fit).

Next, we examined if total external brain area influenced female strength of preference (SOP) for male sexual ornaments throughout the observation period, with SOP treated as a dependent variable that was created using the cbind function, binding “time with high red male” as the success indicator with “time with low red male” as the failure indicator. To avoid overparameterizing this model, we first examined whether the order in which the male dyads were presented affected female SOP scores. To test this, we fit a glmer (as specified above) with SOP as the dependent variable, order of male presentation as a fixed effect, and observation number as a random effect (note that female identity and male dyad identity are not treated as a random effect in this model as their inclusion led to a singularity in the model fit). The order in which male dyads were presented did not affect SOP scores (glmer: z = 0.15, *P* = 0.88) and was therefore not included in subsequent models. To test if SOP scores were affected by female brain size or the male difference in total red area, glmers were run with SOP as the dependent variable (as specified above) with female total relative external brain area, male difference in red area (large vs. small) and their interaction as categorical fixed effects, and female identity and observation number as random effects (note that the model did not converge when including male dyad identity as a random effect and it was therefore removed from the model). To determine if preference deviated from the null expectation of 0.5, we fit a glmer model with the significant categorical effect (in this case the male difference in red area, see Results) against a fixed intercept term and including observation ID as a random effect.

To examine the effects of continuous variation in total relative external brain area and male red area on female preference, we performed a complementary set of glmer models. In a model assessing total brain area, we treated SOP scores (as defined above) as the dependent variable, with the total external brain area, female body length (to account for allometric relationships between brain area and body), and the absolute difference in red area between the two stimuli males included as a covariates and observation number as a random effect. In a model assessing the effect of telencephalon size on female choice, we treated SOP as the dependent variable, with external telencephalon area, the “remaining brain area” (total external brain area—external telencephalon area), and the absolute difference in red area between the two stimuli males included as covariates and observation number as a random effect. Including female identity or male dyad identity in all models examining continuous variation in female brain area or male red area prevented model convergence. Therefore, these random effects were not included in these models.

We also investigated whether total relative external brain area and male coloration influenced female responsiveness (i.e., the amount of time in both male association zones) or the number of switches between stimuli males. Female responsiveness was modeled as a dependent variable in a linear mixed-effect model (lmer) that treated total relative external brain area and male difference in red area (large vs. small) as categorical fixed effects, and female identity as a random effect. Similarly, we investigated whether the number of times a female switched between the two male association zones was influenced by total relative external brain area and male difference in red. The number of switches was log+1 transformed due to a right-skew in the data. As above, a lmer was run with total relative external brain area and male difference in red area (large vs. small) as categorical fixed effects, and female identity and male dyad identity as random effects. Finally, we examined if female responsiveness and the number of switches between males were influenced by continuous variation in total female brain area or telencephalon area, and male red area in lmer models with total external brain area or external telencephalon area, female body length, and the absolute difference in red area between the two stimuli males included as covariates, and female identity and male dyad identity added as random effects. In all models involving female responsiveness, male dyad identity was not included as a random effect as it prevented model convergence.

## RESULTS

### External brain measurements are a valid proxy for internal brain size

There was clear evidence of significant pairwise repeatability between several external and internal brain measures (Table 1, Supplementary Material and Supplementary Figure S1). The highest observed pairwise repeatability was between total external brain area and internal brain weight, followed by total external brain area and internal brain volume (Table 1). While external brain width and internal brain volume were significantly repeatable, the repeatability between external brain width and brain weight was less robust (Table 1). Therefore, total external brain area was used as the best proxy for internal brain weight and volume.

**Table 1:** Linking external and internal measures of brain size

External measure	Internal measure	R [95% CI]	LRT
External brain area	Brain volume	0.56 [0.35, 0.76]	**<0.001**
	Brain weight	0.67 [0.39, 0.79]	**<0.001**
External brain width	Brain volume	0.48 [0.15, 0.69]	**0.003**
	Brain weight	0.40 [0.11, 0.61]	**0.015**
External telencephalon area	Telencephalon volume	0.61 [0.31, 0.90]	**<0.001**
External optic tectum area	Optic tectum volume	0.44 [0.09, 0.69]	**0.007**

The repeatability (R) values for all correlations between external and internal brain measurements, including the 95% confidence intervals within square brackets, are presented. The likelihood ratio test value (LRT) is reported in all cases. All significantly repeatable values are in bold.

External telencephalon area was significantly repeatable with internal telencephalon volume, and likewise external optic tectum area was significantly repeatable with internal optic tectum volume (Table 1). However, as the telencephalon is the brain region most commonly implicated in learning and memory (Bshary et al. 2002; Vargas et al. 2009; for review, see Overmier and Hollis 1990), only telencephalon area was assessed in subsequent analyses.

### Coloration, not brain size, influences female mate preference

**Figure 3:**
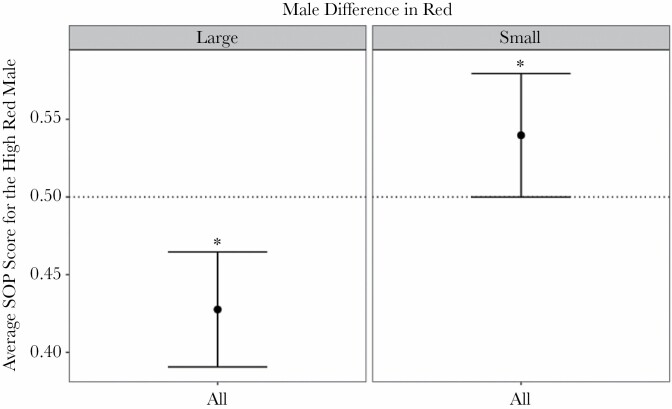
Strength of preference scores. The average strength of preference (SOP) scores for the high red male. SOP scores were calculated as follows: duration of time spent with the high red male/duration of time spent with both males. This allowed for a simple representation of “no choice”: a value of 0.5. Panels are divided by the male difference in red (large/small). 95% confidence intervals of the mean are included. Post-hoc tests revealed that females preferred to associate with males with less red coloration when the difference in sexual ornamentation was large, while females preferred to associate with males with more red coloration when the difference in male sexual ornamentation was small. An asterisk (*) notes a significant difference from 0.5.

Females consistently demonstrated a preference for one of the two males presented in the dichotomous choice assay (z = 12.08, *P* < 0.001). However, total relative external brain area did not influence female strength of preference for males in dichotomous choice trials (Table 2a). Instead, the difference in red area between males (large vs. small) significantly influenced female strength of preference scores (Table 2a), with females preferring to associate with males with less red coloration when the difference in sexual ornamentation was large (z = −2.82, *P* = 0.005), while females preferred to associate with males with more red coloration when the difference in male sexual ornamentation was small, although this was only marginally significant (z = 1.99, *P* = 0.047, Figure 3).

**Table 2:** Brain size, coloration, and female mate preference

Response variable	*N*_females_	Predictors	z	*P*
(a) Large vs. small external total brain area and red coloration				
Female strength of preference	90	Female brain area category (large vs. small)	0.65	0.52
		Categorical difference in red area between males (large vs. small)	**2.92**	**0.004**
		Female brain area × Difference in red area between males	**−**0.70	0.48
(b) Continuous variation in external total brain area and red coloration				
Female strength of preference	90	Female external total brain area	−0.22	0.83
		Female body length	0.83	0.41
		Absolute difference in red area between males	**−3.48**	**<0.001**
(c) Continuous variation in external telencephalon area and red coloration				
Female strength of preference	90	Female external telencephalon area	0.14	0.89
		Female external remaining brain area	0.78	0.43
		Absolute difference in red area between males	**−3.47**	**<0.001**

Outputs of generalized linear mixed-effects models (glmers) investigating how female brain size and the difference in red coloration between male dyads influenced female strength of preference. Female brain size was treated as either a) a categorical variable (large vs. small), determined by assessing the total external brain area relative to body length, b) a continuous variable assessing female total external brain area, or c) a continuous variable assessing female external telencephalon area. Models assessing continuous variation in b) total external brain area or c) external telencephalon area included female body length or the remaining brain area (total external brain area—external telencephalon area) as covariates, respectively, to account for allometric effects. Male difference in red area was treated as a) the categorical (large vs. small) difference in red area between males or b, c) a continuous measure of the absolute difference in red area between males. The total number of females assessed (*N*_females_) is presented for each model, along with the test statistic (z) and *P*-value (p) for each effect. Significant values are in bold.

**Figure 4:**
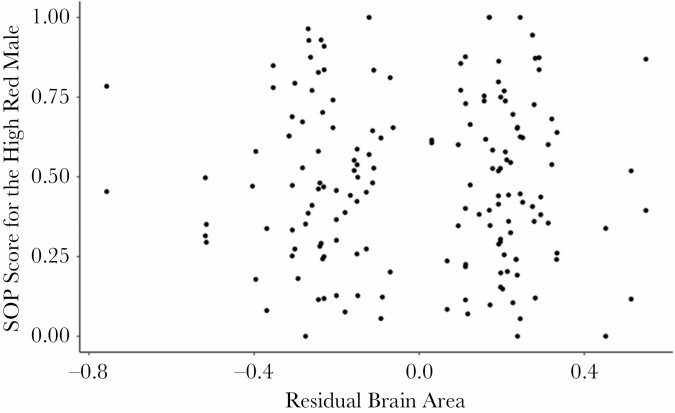
Continuous brain size variation. The effect of continuous brain size variation (standardized using the residual values from a linear regression of external total brain size fitted against standard length) on strength of preference scores for the high red male. SOP scores were calculated as follows: duration of time spent with the high red male/ duration of time spent with both males.

A similar pattern was observed when assessing continuous variation in total external brain area (both total brain and telencephalon area) and when treating the absolute difference between male red coloration as a continuous variable. Specifically, female strength of preference was not influenced by total external brain area (in a model that corrected for allometric effects, Table 2b; Figure 4). However, female preference was negatively related with the absolute difference in red area between males (Table 2b): females demonstrated a preference for less red males when the difference in red between males was large and a weak preference for redder males when the difference between males was small. Similarly, when focusing exclusively on external telencephalon area, female strength of preference was not influenced by external telencephalon area or remaining external brain area, but was negatively related with the absolute difference in red area between males (Table 2c).

Female responsiveness was not affected by total relative external brain area (*t* = 0.27, *P* = 0.79) or the difference in red area between males (large vs. small, *t* = −0.26, *P* = 0.80). Similarly, the number of switches between stimuli males was not affected by total relative external brain area (*t* = 0.79, *P* = 0.43) or the difference in red area between males (large vs. small, *t* = 0.93, *P* = 0.36). We obtained qualitatively similar results when assessing continuous variation in total external brain area and telencephalon area and the absolute difference in red area between stimuli males (Supplementary Table S1).

## Discussion

Contrary to our predictions, variation in female preference is not explained by differences in female brain size relative to body length. Both large- and small-brained females responded similarly to male stimuli, demonstrating preferences that were influenced by the difference in red coloration between male stimuli. This result was unexpected and contradicts two recent studies that have linked brain size, cognitive ability, and mate choice (Corral-López et al. 2017; Rystrom et al. 2019). Instead, we found that socially experienced virgin females preferred males with *less* red coloration when the difference in red coloration between stimuli males was large, presumably making it easier to distinguish between the males. This finding mirrors recent mate choice findings in pygmy halfbeaks which demonstrated that virgin females prefer males with less red coloration (Reuland et al. 2019). In contrast, females preferentially associated with males possessing *more* red coloration when the difference in red coloration between stimuli males was low, although this effect was marginal. Overall, the results of this study highlight that female mate preferences are complex and the link between brain size and mate choice in females is not straight forward.

None of the metrics of female halfbeak brain size that we evaluated in this study influenced female strength of preference scores, responsiveness, or number of switches between stimuli males. Moreover, the relative size of the telencephalon, a brain region responsible for learning and memory (Bshary et al. 2002; Vargas et al. 2009; for review, see Overmier and Hollis 1990) and linked to mate choice decisions in poeciliids (Lynch et al. 2012), was not related to female halfbeak preference. Thus, our study provides no evidence for a link between relative brain size and the expression of mating preferences in halfbeaks. Culumber et al. (2020) reported a similar finding in eastern mosquitofish (*Gambusia holbrooki*), where female success at avoiding male copulation attempts was not influenced by female brain size. Together, these studies suggest that standing variation in brain size may not always explain variation in mating behaviors. In contrast, using artificial selection lines of guppies (*Poecilia reticulata*), Corral-López et al. (2017) reported that large-brained and wild-type females were better able to distinguish between males differing in sexual ornamentation than small-brained females. Interestingly, despite reporting differences in effects between brain size and mate choice, the artificially selected guppies had a comparable variation in relative total brain size between large- and small-brained selection lines compared to the variation observed in large- and small-brained halfbeaks in this study—13.6% difference in brain mass (relative to body size) in guppies (Kotrschal et al. 2013), compared to 11.6% difference in relative external brain area in pygmy halfbeaks.

There are many potential explanations for the emerging disparity in studies assessing the links between brain size and mating behaviors. First, our study and others (e.g., Culumber et al. 2020) use natural variation in brain size to categorize large- and small-brained females. In contrast, Corral-López et al. (2017) used guppies that were artificially selected. Brain morphology is an extremely plastic trait and, in fish, variation in brain size can be influenced by diet, habitat, social environment, and experience (Huber et al. 1997; Kotrschal et al. 1998; Ito et al. 2007; Pollen et al. 2007; Yopak et al. 2007; Kotrschal et al. 2012; Fong et al. 2019). The variation in halfbeak brain size that we exploited in this study may have been the culmination of many different factors acting upon the brain. Consequently, natural phenotypic variation in brain size may not reflect quantitative differences in behavior, highlighting the challenges associated with linking brain morphology to specific performance outcomes. We note that the artificially selected guppy lines with large- and small-brained fish had corresponding high and low numbers of neurons in their brains (Marhounová et al. 2019), while lab populations do not always show correspondence between brain size and neuron number (Herculano-Houzel et al. 2015, but see Kverková et al. 2020). Hence, it would also be valuable to investigate neuron numbers in the halfbeak populations used in this study.

This apparent lack of an effect of brain size on female mate choice in halfbeaks may also be due to the experimental set-up that we used. A dichotomous choice of males may not represent a sufficiently challenging cognitive task for females. In the wild, halfbeaks live in mixed-sex groups ranging from 6 to 128 individuals, which are loosely organized and occasionally shoaling (Greven 2010; Ho et al. 2015; Devigili et al. 2021). This implies that females will consistently be choosing from a large mating pool—a far more difficult choice than that presented in a dichotomous choice chamber. Additionally, constraining social interactions to visual signals in a dichotomous choice chamber prevents a full range of interactions among the animals, which may have minimized the effect of brain size on mating preferences. By attempting to simulate more natural conditions, primarily by providing a choice of more than two mates and allowing free access to males in order to provide additional information such as olfactory cues, effects of brain size on mate preferences may become evident. Thus, allowing free access and a more complex array of signaling to come into play may help to elucidate whether and how brain size influences female mating preferences.

Alternatively, contrary to the assumption of our hypothesis, brain size may not influence mate choice behavior in pygmy halfbeaks. For example, red coloration may be a simple sensory bias signal which triggers an innate response, requiring little cognitive processing (Rodd et al. 2002). This may explain why females with bigger brains did not make “better” or faster choice between males. A larger range of scenarios predicted to be cognitively challenging would have to be tested in order to investigate this.

The main driver of female choice in this study was the difference in stimuli males’ red coloration. Females significantly preferred low red males when the difference between males was large, and high red males when the difference between males was small. Although this appears to be a switch in preference dependent on male difference in red, the post-hoc test examining female preference when the difference in males was small was only marginally significant (z = 1.99, *P* = 0.047). A more parsimonious explanation of the results is that the virgin females used in our study prefer males with less red ornamentation, and this preference is lost when the difference in males is small and the comparison between males is more challenging. This preference for low red males aligns with a previous study in pygmy halfbeaks that found that virgin females (such as those used in this study) prefer males with a small amount of red (Reuland et al. 2019). Reuland et al. (2019) suggested that this preference was due to virgin females’ naivety toward males, rather than their mating status itself. However, females in this study, although virgins, had been previously exposed to males and thus were experienced, suggesting that this preference is innate in virgin females. Generally, female preference for less colorful males is uncommon, although there are some studies which have found a preference for less ornamented males (e.g., house sparrows, *Passer domesticus*, Griffith et al. 1999; European flycatchers, *Ficedula*, Sætre et al. 1997). Interestingly, and contrary to our predictions, both female responsiveness and number of switches between males were unaffected by male difference in red, indicating that females did not seem to invest more time and effort into surveying males with a small difference versus those with a large difference, nor did they differ in their speed at making a decision.

If variation in female halfbeak preference depends on the difference in male red coloration, however, the effect may stem from the salience of red ornamentation and/or female perception of and response to red coloration. Within male dyads with a large difference in red, the average “high red” male had a red area of 0.70 mm^2^, whereas within male dyads with a small difference in red, the average “high red” male had a red area of 0.43 mm^2^. Female halfbeaks may not have been preferentially *associating* with the low red male when the difference in red coloration between male stimuli was large but may have instead been preferentially *avoiding* the high red male. Disentangling active choice from antipathy is challenging in mate choice studies, as both processes can produce similar preference values (Rosenthal 2017). Describing the shape of the female halfbeaks’ preference function for red coloration would help to clarify the relative importance of choice and antipathy in halfbeak female mating preferences.

In conclusion, this study confirms earlier reports (Reuland et al. 2019) of unexpected patterns of mate choice in female halfbeaks. Uncovering the proximate mechanisms driving female preference for either high or low red coloration in males is an important next step for understanding female mating decisions in halfbeaks. Yet, contrary to previous research (Corral-López et al. 2017), this study found that both total brain size and telencephalon size had no effect on mate choice decisions in the pygmy halfbeak. This was unexpected, as brain size is often linked to cognitive ability (Sol et al. 2005; Kotrschal et al. 2013; van der Bijl et al. 2015), and cognitive ability is intuitively and empirically (Rystrom et al. 2019) linked to mate choice decisions. In order to understand the contradicting effects of brain size on mate choice between pygmy halfbeaks and guppies (Corral-López et al. 2017), it would be useful to better characterize the ecological and social setting of halfbeaks in order to compare them to the better-known guppy. Overall, this study offers no evidence that brain size plays a role in driving variation in female preferences in halfbeaks. Whether halfbeaks alter their mate choice decisions based on observing individuals perform cognitively demanding tasks, as recently demonstrated in other species (Chen et al. 2019; but see Keagy et al. 2019), remains to be tested. Nevertheless, the simple technique of measuring brain size externally in halfbeaks described here opens up a wide avenue for future study assessing whether the variation in brain size found in halfbeaks affects their performance in other cognitively challenging tasks (e.g., numerical learning assays, Agrillo et al. 2007; Kotrschal et al. 2013; spatial learning tasks, Elias 1969; Lewejohann et al. 2010; Kotrschal, Corral-Lopez, et al. 2015; and survival rates under predation threat, Sol et al. 2007; Kotrschal, Buechel et al. 2015; van der Bijl et al. 2015). Ideally, future studies should be carried out on individuals in the field, as the potential effect of laboratory conditions on the relative brain size of pygmy halfbeaks is unknown. In addition, the use of free-swimming assays instead of a dichotomous choice chamber may better elucidate brain size effects (for a review of mate choice experimental designs, see Dougherty 2020). Gaining a clearer understanding of the link between cognitive ability and brain size will help to better contextualize the role of brain morphology in influencing mate choice decisions.

## Supplementary Material

arab046_suppl_Supplementary_Data

## Data Availability

Analyses reported in this article can be reproduced using the data provided by McNeil et al. (2021).
