# Supplementary material for: Does brain size affect mate choice? An experimental examination in pygmy halfbeaks
Source: Behav Ecol. 2021 Aug 23;32(6):1103–13. doi: 10.1093/beheco/arab046 (PMC8691582; doi:10.1093/beheco/arab046)
Supplement: arab046_suppl_Supplementary_Data [file arab046_suppl_supplementary_data.doc]

# Does brain size affect mate choice? An experimental examination in pygmy halfbeaks

Rebecca M. McNeil, Alessandro Devigili, Niclas Kolm, and John L. Fitzpatrick

**Supporting material**

**Supporting methods and results**

We assessed the strength and direction of correlations among body length and all external and internal brain measurements. A correlation plot was generated using the ‘pairwise.complete.obs’ function in the R package ‘corr.plot’ (Wei and Simko 2017) which computes pearson’s correlation coefficients for all pairs of complete observations for every relevant measure in the log-transformed dataset. Significance levels were set to 0.05. Overall, brain measures were highly correlated (Figure S1). The correlation values give similar conclusions to the repeatability values – external brain area is significantly and highly correlated with internal brain volume and weight, whereas external brain width is a weaker correlate of internal brain volume and weight. External brain length, in comparison, does not correlate with internal brain measures. In addition, external optic tectum area and internal optic tectum volume are weakly but significantly correlated. External telencephalon area is highly correlated with internal telencephalon volume, further motivating its use as a proxy for telencephalon volume. External brain area (the chosen proxy for brain size) also correlates well with many individual brain areas – telencephalon volume, optic tectum volume and hypothalamus volume. However, it does not significantly correlate with cerebellum volume, dorsal medulla volume and olfactory bulb volume.

**Figure Legends**

**Figure S1**: Correlations between female body size, external brain measures and internal brain measures. Female body size was measured using standard length. External brain measures include total brain area, telencephalon area, optic tectum area and brain length and width. Internal brain measures include total brain weight, total brain volume, and the volume of individual brain regions (telencephalon, optic tectum, cerebellum, dorsal medulla, olfactory bulbs and hypothalamus). All variables were log-transformed. An empty box indicates a non-significant correlation. All significant correlations were positive as can be seen from the absence of red circles. Circle size and darkness of blue indicates the strength of the significant correlations.

**Table S1**: **Brain size, colouration and female responsiveness and switches between stimuli males.** Outputs of linear mixed-effects models (lmers) investigating differences in female responsiveness and number of switches when assessing a) continuous variation in external brain area relative to body size and red colouration b) continuous variation in external telencephalon area relative to total brain area and red colouration. All models included female body length as a covariate to account for allometric effects. The total number of females assessed (N_females_) is presented for each mode, along with the degrees of freedom (df), test statistic (z) and p-value (p) for each effect. Models included female identity and male dyad identity as random effects.

**Table S1**

| **Response Variable** | | **N_females_** | **Predictors** | **df** | ***t*** | **p** |
| --- | --- | --- | --- | --- | --- | --- |
| ***a) Continuous variation in external total brain area and red colouration*** | | | |  |  |  |
|  | Female responsiveness | 90 | Female external brain area | 84.06 | -0.33 | 0.74 |
|  |  |  | Female body length | 85.67 | -0.86 | 0.39 |
|  |  |  | Male difference in red | 96.64 | 0.23 | 0.82 |
|  |  |  |  |  |  |  |
|  | Female number of switches between males | 90 | Female external brain area | 80.08 | -0.80 | 0.43 |
|  |  |  | Female body length | 83.71 | 0.90 | 0.37 |
|  |  |  | Male difference in red | 34.35 | -1.34 | 0.19 |
|  |  |  |  |  |  |  |
| ***b) Continuous variation in external telencephalon area and red colouration*** | | | |  |  |  |
|  | Female responsiveness | 90 | Female external telencephalon area  Female external remaining brain area | 81.48  81.80 | -0.02  -1.27 | 0.93  0.21 |
|  |  |  | Male difference in red | 96.59 | 0.24 | 0.82 |
|  |  |  |  |  |  |  |
|  | Female number of switches between males | 90 | Female external telencephalon area  Female external remaining brain area | 80.29  80.47 | 0.51  -0.42 | 0.61  0.67 |
|  |  |  | Male difference in red | 34.94 | -1.36 | 0.18 |
|  |  |  |  |  |  |  |

**Supplementary references**

Wei, T. & Simko, V. 2017. R package "corrplot": Visualization of a Correlation Matrix (Version 0.84).
